# Threshold Electric Skin Sensitivity Fluctuations in Pregnancy, Labor, and Puerperium

**DOI:** 10.1089/bioe.2019.0011

**Published:** 2020-03-18

**Authors:** Charles Boris Cernoch Rossmann, William von Taaffe, Cosima von Taaffe

**Affiliations:** ^1^ Department of Obstetrics and Gynecology, Western Memorial Regional Hospital, Corner Brook, Canada.; ^2^ Department of Internal Medicine, Morton Plant Hospital, Clearwater, Florida.; ^3^ Western Memorial Regional Hospital, Corner Brook, Canada.

**Keywords:** threshold electric skin sensitivity measurement, referred pain dermatomes, irritable bowel syndrome, intestinal motility, pregnancy

## Abstract

***Hypothesis:*** In our clinical study, the stages of pregnancy were independent variables, and the measured change of sensitivity in selected skin areas was the dependent variable. The research hypothesis that the threshold electric skin sensitivity (TESS) fluctuates was based on our previously published research, where the null hypothesis was rejected in similar conditions.

***Methods:*** TESS was measured repeatedly in short intervals on the abdomen and right forearm of pregnant and not pregnant women.

***Results:*** A statistically very significant change of TESS fluctuation was found between not pregnant and pregnant women in different stages of pregnancy, labor, and postpartum. In the midline above the navel, the TESS fluctuates with similar frequency like intestinal peristalsis and that may reflect on the functional state of the bowels. In both lateral areas of the lower abdomen in not pregnant women and in early pregnancy, we found only minimal TESS fluctuations. They gradually increased until the beginning of labor and then decreased postpartum. In midline above the pubic symphysis, moderate TESS fluctuation in not pregnant women gradually increased during pregnancy, until the beginning of labor and decreased postpartum. TESS fluctuation during labor was not synchronous with recorded uterine contractions. Only minimal TESS fluctuation on the right forearm never changed significantly.

***Conclusion:*** TESS fluctuation in midline abdomen may be related to bowel peristaltic motility. In the lateral abdomen and above symphysis, it may be related to uterus activity. TESS measurement in referral pain skin area could be used for functional monitoring of internal organ in the corresponding viscerotome. The measurement of TESS in skin areas and recording fluctuations graphically as an electrosensitogram, could have diagnostic value. As a new noninvasive diagnostic method, electrosensitography could help us better understand the gastrointestinal organ function.

## Introduction

The Threshold Electric Skin Sensitivity (TESS) was intensively investigated by many researchers during the past 100 years and a good survey of the previous literature is discussed in the work of Notermans.^1^ He used square wave constant current stimulus and tried to determine how many variables could have an influence on the skin sensitivity to electric stimulation. He found nearly constant pain threshold when measured in the same individual over a course of time. Schumacher et al.^2^ measuring threshold sensitivity to heat stimulation in an earlier study came to the same conclusion. On the contrary, Lanier^3^ found that the threshold sensitivity to electric skin stimulation could vary considerably.

Most authors measured the skin sensitivity in many different skin areas, but not repeatedly in short intervals over the same area of the same subject. Uher et al.^4^ found decreased electric skin sensitivity above the pubic symphysis in early labor and after the administration of oxytocin. He found increased sensitivity after administration of strychnine. In a previous study, Cernoch et al.^5^ have used the same stimulator with constant voltage square waves on pregnant and puerperal women and found long-term changes of skin sensitivity. Later, he also found interesting short-term changes of sensitivity when he used a stimulator with constant current square waves on puerperal women after administration of oxytocin and neostigmine.^6^ In pregnant women, he found significant skin sensitivity changes after intravenous administration of oxytocin already before the beginning of the uterine contraction. We tried to find under the same conditions, changes in other biophysical parameters of the skin; however, measurement of the skin temperature and electric conductivity did not reveal any similar changes. Cupr et al.^7^ were using the same constant current square wave stimulator constructed by Valosek and Salansky.^8^ They found different skin sensitivity during and between the uterine contractions in laboring women with a history of dysmenorrhea and no change with no dysmenorrhea.

As Cernoch et al.^5,6^ found in the previous investigations, there are long-term and the short-term changes of TESS in specific areas of the abdomen during pregnancy, labor, and puerperium. During labor, average TESS above the pubic symphysis and in the lateral areas of the lower abdomen is increased, but around the navel it is decreased. After administration of oxytocin to puerperal women, the usually steady TESS in the lateral abdomen suddenly starts fluctuating up and down. After administration of neostigmine, there is no change of steady TESS in lateral areas, but there is a prominent increase of TESS fluctuations around the navel and above the pubis. These TESS fluctuations may be related to the functional state of the internal organs such as the uterus and bowel. The dermatome skin area for the uterus is on both sides of the lower abdomen, fusing together above the pubic symphysis as a dermatome for the uterine cervix.^9^ The dermatome skin area for the bowel is in the midline of the abdomen.^10^

## Materials and Methods

The TESS was measured repeatedly in short intervals on the abdomen and right forearm of 20 not pregnant and 50 pregnant women. The subjects were informed of the measurement procedure and they gave informed consent to participate in the study. The mean age was 23 years (range 15–43 years). They were randomly selected from available patients who came to the clinic and hospital for treatment by Dr. Rossmann and Rossmann.^11^

We used in this study a different stimulator, than that in the previous research, because the previously used stimulator was no longer available. It was also easier to use the NS-3 peripheral nerve stimulator from Professional Instruments Co., Houston, TX. It produces a rectangular single pulse of 0.2-ms duration in 1-s intervals. The nerve stimulating current can be regulated from 0 to 20 mA, and the output voltage can be read from the scale as a data. Increasing the applied voltage at constant skin impedance increases the electrical current that reaches the skin sensory receptors.

**FIG. 1.**
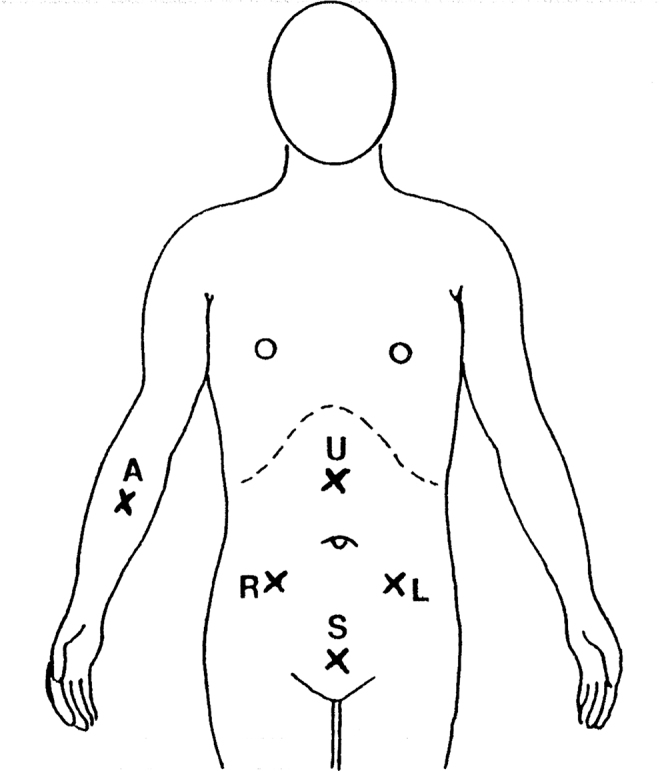
Location of stimulating electrodes on abdomen and forearm: R, right lower abdomen; L, left lower abdomen; U, midline above umbilicus; S, midline above symphysis; A, right forearm.

As a stimulating electrode, we used disposable Red Dot 3M electrodes with solid gel and Micropore R tape. Four electrodes were placed on the abdomen and on the right arm (Fig. 1). As a grounding electrode, we used a disposable NDM Dia Temp II self-adhering pad placed on the right thigh. Each subject would lie relaxed on the bed or on the examination table and disposable electrodes were then attached to the skin. Before starting the measurement, the patient was instructed to immediately say “yes” when she started to feel the first sensation below the electrode. Next, a few orientation stimulations were done to make the patient familiar with the procedure and to exclude effect of learning on the results. While working as an OBGYN specialist in Newfoundland, Canada, I have done on 70 volunteer patients ∼30,300 single TESS measurements during 280 sessions, each lasting 15 to 30 min. During each session, I measured TESS in episodes lasting 3 to 5 min on each of the five electrodes. The sequence of measurements on electrodes was as follows: (1) right side of the abdomen, (2) left side of the abdomen, (3) middle above umbilicus, (4) middle above pubic symphysis, and (5) right forearm. During each of the episodes, I did the single TESS measurement repeatedly in 5- to 10-s intervals. During labor, the patient's uterine contractions were simultaneously recorded by a Hewlett-Packard cardiotocography fetal monitor. Uterine contractions and TESS fluctuations were then transcribed in the same graphic record for easy visual correlation. The length of TESS measurements during labor was limited by discomfort and the wiliness of the patient to cooperate. The patient did not receive any analgesia before the measurement.

**FIG. 2.**
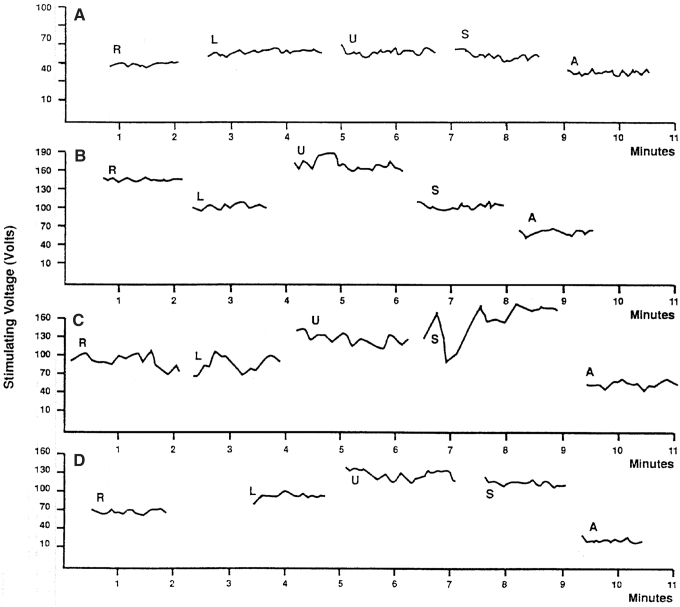
Examples of ESG graphs: **(A)** nonpregnant women, **(B)** at 29th week of pregnancy, **(C)** at 39th week of pregnancy, **(D)** postpartum. Location of electrodes: R, right lower abdomen, L, left lower abdomen, U, midline above umbilicus, S, midline above symphysis, A, right forearm. ESG, electrosensitogram.

The TESS measured data were dictated into a tape recorder, transcribed in tables as a function of time, and plotted on the graph. To express the TESS fluctuation phenomenon in score data, the length of the curve line was measured in millimeters and divided by the time of measurement in minutes (mm/min). The higher score represents fluctuations of bigger amplitude or frequency. Later, we started using a simple method for expressing the TESS fluctuations in score data. We calculated the standard deviation for values of TESS collected in each measurement episode. The value of the standard deviation used as a fluctuation score data closely correlates with the fluctuation score data received by measuring the length of the plotted sensitivity curve per minute. Only score data obtained this way were used for further statistical analysis and for presentation in this report. The measured TESS data were also plotted as a function of time in the graph. Figure 2 shows examples of a typical electrosensitogram (ESG) in nonpregnant, pregnant, and postpartum women.

In the present study, independent variables included pregnant or postpartum patients divided into groups according to gestational age, irregular or regular contractions during labor, and days postpartum. For all the statistical analyses presented in this report, only two levels of the independent variable were used in one-way, between-subject's analysis of variance (ANOVA). The dependent variable is the calculated TESS fluctuation score data. Fluctuation score data equal the standard deviation for the collected sensitivity data during one measurement episode. The alfa significance level was selected at 0.05. Each time we found a statistically significant difference between the two levels of independent variables, the strength-of-association measure test was done using the eta-squared computational formula.

## Results

**FIG. 3.**
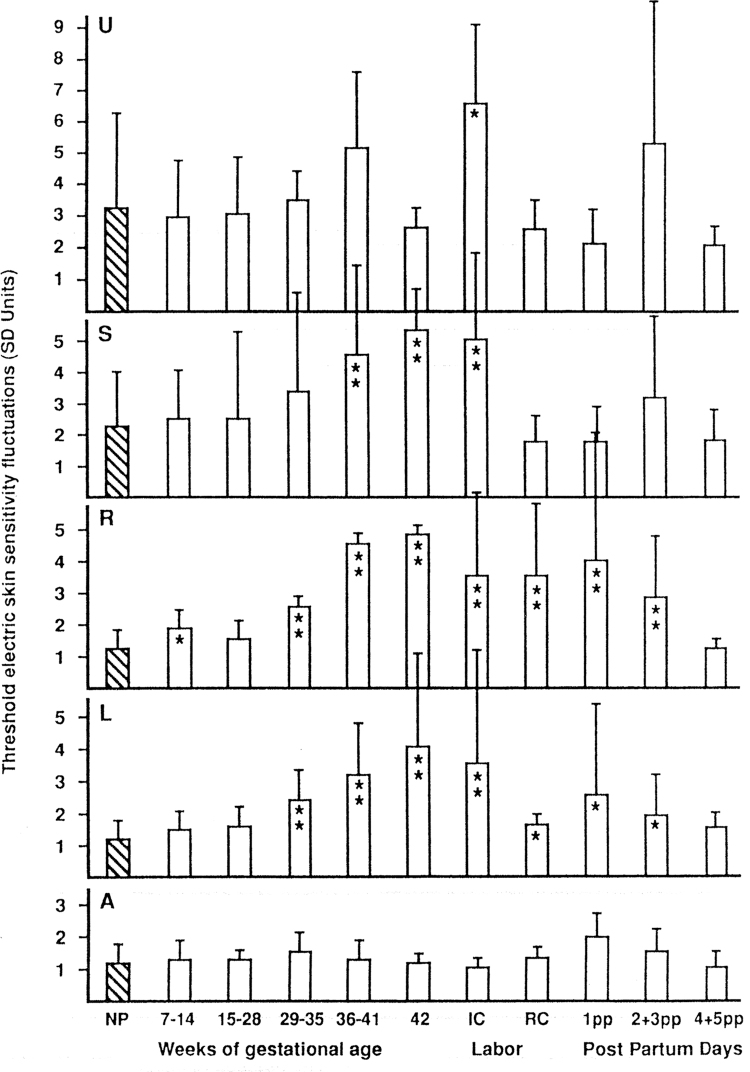
Changes of threshold electric skin sensitivity fluctuations during pregnancy, labor, and postpartum. PANELS: U, midline above umbilicus; S, midline above symphysis; R, right lower abdomen; L, left lower abdomen; A, right forearm. BARS: NP, not pregnant, 7–14, 15–28, 29–35, 36–41, 42, weeks of gestational age; IC, irregular contractions; RC, regular contractions; 1pp, 2 + 3pp, 4 + 5pp, days postpartum. Values statistically significant are indicated by *p < 0.05 or **p < 0.01.

Figure 3 illustrates fluctuations of TESS in five different areas of the skin of the abdomen and right forearm. The height of the bars indicates the mean intensity of TESS fluctuations in groups of women who are not pregnant, or in consecutive stages of pregnancy and postpartum. Above the navel, the sensitivity fluctuates most of the time. The only statistically significant change (*p* < 0.05) is between nonpregnant and pregnant women with irregular contractions. There is a decrease of TESS fluctuations when contractions become regular during labor and postpartum. Above the pubic symphysis, the TESS fluctuations gradually increase during the pregnancy; but similarly, like above the navel, there is a great variability. Only increases of TESS fluctuations from 36 weeks of pregnancy to the beginning of the irregular contractions of early labor are statistically very significant (*p* < 0.01). During regular contractions of labor and postpartum, there is a marked decrease of TESS fluctuations.

TESS of the lateral skin areas of the abdomen differs from that of the medial areas. There is consistently only minimal fluctuation of TESS in nonpregnant women and in early pregnancy. However, statistically, there is a very significant increase (*p* < 0.01) of TESS fluctuations from the 29th week of pregnancy to the beginning of irregular contractions. In most of the women, postpartum TESS fluctuations decrease in lateral areas; however, a few of them had considerable TESS fluctuations resulting in a large variability in measured data. The control skin area on the right forearm demonstrated consistently minimal fluctuations of TESS, and no statistically significant changes between all groups of patients were found. It is interesting that from the fourth day postpartum, the TESS fluctuations in all areas returned to the values of not pregnant women. In contrast, there was a large variability of TESS on the third day after labor, especially above the navel.

**FIG. 4.**
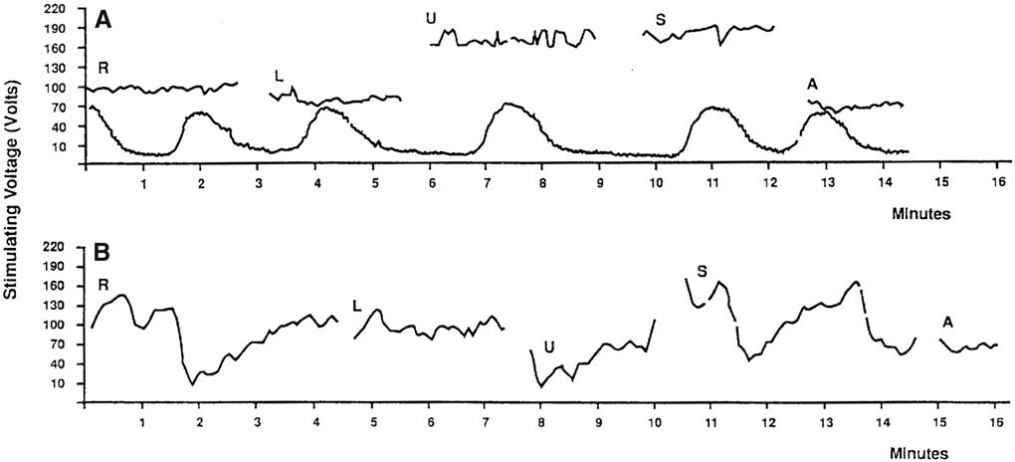
**(A)** Tocogram recorded simultaneously with ESG during labor. **(B)** ESG of patient with mild preeclampsia at 38 weeks of gestational age.

In 10 women, the contractions were recorded simultaneously during the skin TESS measurement. As seen in Figure 4A, the tocographic record has been transposed on the skin sensitivity graph so that visual correlation can be done between both curves. There is no similarity between the two curves. We did not find so far, any practical use of ESG in obstetrics for monitoring uterine contractions. We found that women in labor with irregular contractions have more TESS fluctuations than later during the regular uterine contractions of labor. The means for the levels of the independent variables are reported in Table 1. The means for ANOVA performed on these data indicated that there were statistically significant differences (*p* < 0.05) among the means for the factors presented in Table 2. The null hypothesis must therefore be rejected, and the conclusion reached was that the fluctuations of TESS change in certain skin areas of the abdomen during pregnancy, labor, and postpartum. The strength-of-association measure (eta-square) for the statistically significant changes with *p* < 0.05 was between 11% and 22%. The eta-square for statistically very significant changes with *p* < 0.01 was found between 26% and 62%. It indicates that in these situations there is a very strong relationship between the independent and dependent variables and provides justification for making strong inferences about the validity of the presented results.

**Table 1.** Changes in Electric Skin Sensitivity Fluctuations During Pregnancy, Labor, and Postpartum

Measured in area	Parameter	Not pregnant	Pregnant week 7–14	Pregnant week 15–28	Pregnant week 29–35	Pregnant week 36–41	Pregnant week 42	Contractions not regular	Contractions regular	Post partum day 1	Post partum day 2 + 3	Post partum day 4 + 5
Middle above umbilicus	Mean	3.2	3.0	3.1	3.4	5.1	2.7	6.6	2.5	2.1	5.2	2.0
	SD	3.1	1.8	1.8	0.9	2.5	0.6	2.6	0.9	1.1	5.6	0.6
	*N*	19	25	5	6	11	4	7	4	4	9	5
Middle above symphysis	Mean	2.2	2.6	2.7	3.4	4.8	5.3	5.1	1.8	1.8	3.2	2.0
	SD	1.8	1.5	2.7	3.2	2.7	1.2	2.6	0.7	0.8	2.7	0.8
	*N*	19	25	5	6	11	4	7	4	4	9	3
Right lower abdomen	Mean	1.2	1.9	1.4	2.7	4.5	4.8	3.4	3.5	4.1	2.9	1.2
	SD	0.6	1.3	0.3	2.1	2.8	2.6	2.4	3.3	4.1	2.0	0.4
	*N*	19	25	5	6	11	4	7	4	4	9	3
Left lower abdomen	Mean	1.2	1.5	1.5	2.3	3.2	4.2	3.6	1.7	2.6	1.0	1.6
	SD	0.5	0.6	0.7	0.8	1.7	2.9	3.5	3.6	3.0	1.2	0.5
	*N*	19	25	5	6	11	4	7	4	4	9	5
Right forearm	Mean	1.3	1.4	1.3	1.7	1.5	1.2	1.1	1.3	2.1	1.6	2.1
	SD	0.6	0.5	0.1	0.6	0.5	0.3	0.3	0.4	0.8	0.8	0.5
	*N*	20	23	3	5	10	3	6	4	3	6	4

Mean, mean threshold electric skin sensitivity fluctuations; *N*, number of measured subjects; SD, standard deviation from mean.

**Table 2.** One-Way Between-Subjects Analysis of Variance of Threshold Electric Skin Sensitivity Fluctuations

Source	df	SS	MS	F	Eta-square (%)	p	Significance
Factor R:7–14 GA	1	5.3	5.3	5.01	11	<0.05	^*^
Error	42	44.7	1.1				
Factor R:29–35 GA	1	10.7	10.7	8.51	27	<0.01	^**^
Error	23	29.1	1.3				
Factor L:29–35 GA	1	5.5	5.5	17.25	43	<0.01	^**^
Error	23	7.3	0.3				
Factor S:36–41 GA	1	46.7	46.7	9.88	26	<0.01	^**^
Error	28	132.5	4.7				
Factor R:36–41 GA	1	76.1	76.1	24.53	47	<0.01	^**^
Error	28	86.9	3.1				
Factor L:36–41 GA	1	28.4	28.4	25.14	47	<0.01	^**^
Error	28	31.4	1.1				
Factor S:42 GA	1	32.6	32.6	10.98	34	<0.01	^**^
Error	21	62.4	3.0				
Factor R:42 GA	1	42.8	42.8	34.42	62	<0.01	^**^
Error	21	26.1	1.2				
Factor L:42 GA	1	29.1	29.1	20.75	50	<0.01	^**^
Error	21	29.5	1.4				
Factor U: IC	1	60.3	60.3	6.86	22	<0.05	^*^
Error	24	210.9	8.8				
Factor S: IC	1	44.4	44.4	10.69	31	<0.01	^**^
Error	24	99.6	4.2				
Factor R: IC	1	24.9	24.9	14.78	38	<0.01	^**^
Error	24	40.4	1.7				
Factor L: IC	1	30.8	30.8	9.53	28	<0.01	^**^
Error	24	77.6	3.2				
Factor R: RC	1	18.0	18.0	16.93	45	<0.01	^**^
Error	21	22.3	1.1				
Factor L: RC	1	0.9	0.9	4.70	18	<0.05	^*^
Error	21	4.2	0.2				
Factor R: 1pp	1	27.6	27.6	10.23	33	<0.01	^**^
Error	21	56.7	2.7				
Factor L: 1pp	1	7.0	7.0	4.86	19	<0.05	^*^
Error	21	30.1	0.3				
Factor R: 2 + 3pp	1	17.8	17.8	11.71	31	<0.01	^**^
Error	26	39.5	1.5				
Factor L: 2 + 3pp	1	4.3	4.3	7.67	22	<0.05	^*^
Error	26	14.7	0.6				

^*^ Significant *p* < 0.05.

^**^ Very significant *p* < 0.01.

df, Degree of freedom; *F*, F test value; GA, gestational age, weeks; IC, irregular contractions in labor; L, left lower abdomen; MS, mean square; *p*, statistical significance; R, right lower abdomen; RC, regular contractions in labor; S, midline above symphysis; SS, sum of squares; U, midline above umbilicus.

Measurements were also done on six patients with preeclampsia, severe gestational edema, acute ureteral obstruction, and irritable bowel, which did show especially large TESS fluctuations in most of the measured areas. However, there were too few patients with these conditions measured to make further analysis at this time. Typical ESG in mild preeclampsia is shown in Figure 4B.

**FIG. 5.**
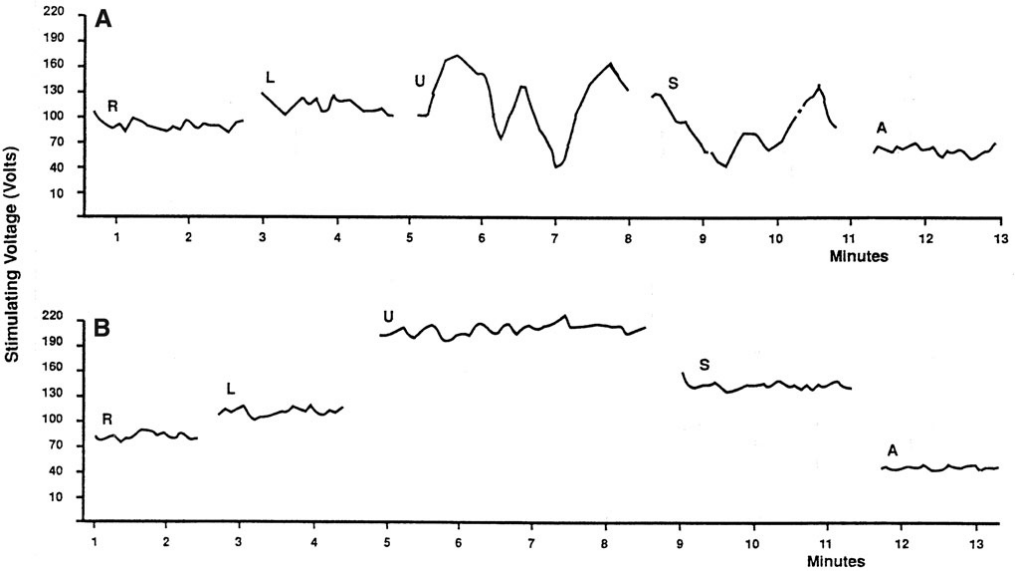
**(A)** ESG of a 34-week pregnant patient with acute functional ureteral obstruction. **(B)** The same patient 4 days after the obstruction was relieved by a ureteral stent.

Similar, large TESS fluctuations were found in one 34-week pregnant patient with acute functional ureteral obstruction. After the obstruction was relieved by a ureteral stent, the TESS fluctuations gradually decreased over several days and this is significant. However, the increased TESS fluctuations could be related to increased gastrointestinal motility (Fig. 5).

## Discussion

Our present work supports the research hypothesis that there are changes of short-term TESS fluctuations during consecutive stages of pregnancy, labor, and postpartum. Maximal TESS fluctuations were found in all measured skin areas during late pregnancy and during irregular contractions at the beginning of labor. During regular uterine contractions, however, the TESS fluctuations decreased, and we could not confirm our research hypothesis that there would be some temporal relationship between the TESS (ESG) curve and the tocographic record of the uterine contraction. Minimal TESS fluctuations were generally found in most of the skin areas of nonpregnant women and this served as a control independent variable. Increased TESS fluctuations during late pregnancy and labor are consistent with our previous research findings, when we administered oxytocin and neostigmine to puerperal women. This could be related to increased intestinal motility caused by oxytocin. The underlying neurophysiological mechanism, however, is not clear.

As we postulated in previous articles,^5,6^ the skin sensitivity fluctuations in the midline of the abdomen may be related to the functional state (motility) of the bowels. If any such relationship could be proven by further research, for example, by performing simultaneously ESG and colon manometry, it could have practical applications, such as in gastroenterology, as a noninvasive diagnostic method for screening and differential diagnosis of gastrointestinal motility disorders. Iovino et al.^12^ found in irritable bowel patients, somatic hypoalgesia to electrical stimuli.

The fluctuations of TESS in the lateral abdominal areas and above the pubic symphysis are more difficult to explain. Oxytocin and labor clearly have a significant effect on it; but we found that the changes were not synchronous with the uterine contractions. On the contrary, we found in our previous work that TESS fluctuations on the right- and left-side of the abdomen change synchronically after giving oxytocin to puerperal women.^6^ In the present study, the subjects did not receive any medication.

The TESS fluctuations in the lateral areas of the abdomen may be related to the activity of the autonomic nervous system in the uterus. If this is true, the change of TESS in dermatomes would be an expression of the autonomic activity of the internal organs in the corresponding viscerotome. Because there is an overlapping between the viscerotome of the lower gastrointestinal tract and viscerotome of the internal genital system in women, the interpretation of the ESG may be difficult. Our statistically very significant results indicate that it is possible. The anecdotal findings of TESS fluctuation changes in pregnant patients with ureteral obstruction, preeclampsia, and irritable bowel syndrome may be of interest for further studies in these and other clinical situations. Another improvement would be combining TESS measurements with an objective method to monitor subject's response to quantitative stimuli.^13^ In the previous study, we used a constant current stimulator,^8^ based on Laufberger's excitation theory, using two electric current impulses of different lengths and intensity.^14^

## Conclusion

In our investigation, we found that TESS fluctuates in different areas of the abdominal skin and that these fluctuations also change during pregnancy, labor, and postpartum. Clinicians are using the manifestations of referred pain in dermatome to help diagnose pathological and physiological processes of the internal organs in the corresponding viscerotome inside the human body. Similarly, the exact measurement and recording of TESS in dermatome could have a diagnostic value in bowel motility disorders such as irritable bowel syndrome.

As a noninvasive method, it could help better understand the function of involved internal organs in the corresponding viscerotome.
